# Strategies for recruiting the dependent children of patients with a life-limiting illness as research participants

**DOI:** 10.1177/02692163221122302

**Published:** 2022-09-14

**Authors:** Steve Marshall, Pam Stephenson, Denice Sheehan

**Affiliations:** 1 Cicely Saunders Institute of Palliative Care, Policy & Rehabilitation, Florence Nightingale Faculty of Nursing, Midwifery & Palliative Care, King’s College London, London, UK; 2 Kent State University College of Nursing, Kent, OH, USA

**Keywords:** Child, adolescent, family, parents, bereavement, critical illness, qualitative research, patient selection

## Abstract

**Background:**

The voices of children and adolescents have historically been substituted by the perspective of adults. There is growing recognition that children (<18 years old) are able to participate in research and appreciate the opportunity to participate in studies.

**Aim:**

To share the strategies employed by two research teams from USA and UK, who have successfully recruited children living with parental life-limiting illness as research participants.

**Findings:**

The researchers overcame common challenges when negotiating ethics committees by anticipating the IRB/REC concerns, providing the committees with detailed applications including distress protocols, and offering resources to their ethics committee to learn about conducting research with this population. The researchers navigated recruitment and gatekeeping by clinicians and parents by partnering with clinical agencies and nurturing relationships with practitioners who are supportive of the research, offering to present the findings of the study with continuing education credits, and developing children’s, adolescents’ and parents’ project advisory groups to support recruitment, data collection and analysis.

**Conclusions:**

Simple strategies can be used to overcome barriers to recruitment, providing opportunities for children to be research participants and for their unique perspectives to be heard in palliative care research.

**What is already known about the topic?**

- The voices of children are under-represented in palliative care research.
- Children appreciate participating and have the right to be involved in palliative care research.
- In order to include children, researchers have to negotiate complex ethical issues and navigate many barriers to recruitment.

**What this paper adds?**

- The main challenges to recruiting children as research participants were found to be: negotiating ethics committees and navigating gatekeeping in recruitment.
- The paper offers strategies to negotiate ethics committees.
- The paper provides facilitators to navigate gatekeeping in recruitment.

**Implications for practice, theory or policy**

- In order to fulfil their right to be involved in all matters affecting them, children should be involved in palliative care research.
- Researchers should consider creative strategies to ensure that the voices of children are included in palliative care research.
- Ethics committee members, clinicians and parents require education about the importance of including children in palliative care research.

## Background

Traditional research methodologies have tended to substitute the perspective of adults as proxies for the voices of children (<18 years old).^1^ Founded on the principle that children have the right to have a say in all matters affecting them, the United Nations (UN) Convention on the Rights of Children^2^ has been influential in encouraging children’s active participation in all aspects of the research process.^3,4^ There is a growing recognition that children have agency, are able to construct their own meanings and with appropriate support from researchers can articulate their unique perspective.^5,6^ The contribution of children has the potential to improve research and ensure that evidence-based policy or services accurately reflect children’s lived realities.^1,7^

Recruiting children as research participants can be challenging and researchers have to navigate around complex ethical issues including confidentiality, informed consent, parental consent, children’s assent, privacy and risk-benefit to participants.^8,9^ There can be additional challenges when the research occurs in palliative care, due to the sensitivity of the topic and the perception that these children are especially vulnerable.^10,11^ Although there are distinct challenges when the subject of the research is children with serious illnesses, as opposed to the dependent children of a parent with a serious illness, the complexities of conducting any research with children can be discouraging to researchers and the voices of children are therefore under-represented in the field of palliative care.^12,13^

However research with this cohort is possible and an increasing number of palliative care studies across the world include the voices of children.^14–20^ The authors are from two research teams, one in the United Kingdom (UK) and one in the United States (USA), which have successfully recruited the dependent children of patients with a life-limiting illness to participate in qualitative research. Despite the differences in how health services are structured in each country, both teams experienced common challenges to conducting research with this cohort. The two teams also found that they had employed similar strategies to overcome these challenges and enable the perspective of children to be represented in palliative care research.

## Aim

To share the strategies employed by two research teams from USA and UK, who have successfully recruited children living with parental life-limiting illness as research participants.

## Design/setting/participants

The research teams individually recruited children to the following studies:

- Semi-structured interviews were conducted with nine parents with advanced cancer enrolled in a hospice program, seven of their spouses/partners and 10 of their children aged 13–17 (USA).^17^ Recruitment was achieved with the support of hospice staff, who introduced the study to potential participants and obtained consent for the research team to make contact. The number of refusals was not recorded.
- Semi-structured interviews were conducted with 14 ill parents enrolled in a hospice program, 17 of their spouses/partners and 30 of their children aged 13–17.^19^ Semi-structured interviews were subsequently conducted with six of the children from this study, 2–13 months after the parent’s death (USA).^21^ Recruitment was achieved by promoting the study regularly with hospice staff. An additional 19 families met the eligibility criteria, but did not participate because of the following: no reason provided (*n* = 11); parent died before enrolment (*n* = 4); family were not interested (*n* = 2); child was unaware their parent was seriously ill (*n* = 1); and the family was conflicted about the child’s participation (*n* = 1).
- Semi-structured interviews were conducted with 32 children aged 6–17, whose parent was living with life-limiting illness (UK).^22^ Participants were recruited by extensively and repeatedly promoting the study with hospital teams, hospices, on social media and via voluntary organisations. An additional 16 participants were identified or self-selected, but did not participate because: contact was lost (*n* = 8); child decided not to participate (*n* = 3); parent decided against participation (*n* = 2); child was not eligible to participate (*n* = 2); and parent died prior to interview (*n* = 1).

Each study adhered to established qualitative methods for data collection and analysis,^23^ including independent coding followed by regular team meetings and consensus building to identify emerging findings. Many of the points covered by this paper are the result of field notes that were created and reviewed by members of each research team.

## Results

The challenges to recruiting children as research participants were found to be around two main areas: negotiating ethics committees and navigating gatekeeping in recruitment.

### Negotiating ethics committees

In the USA all human subjects research must be reviewed by and receive approval from an Institutional Review Board (IRB), which are maintained by individual universities and hospitals. In the UK research within the National Health Service (NHS) must obtain approval from an NHS Research Ethics Committee (REC) and simultaneously be approved by a host university. The common challenges faced when seeking to obtain IRB or REC ethical approval are: resistance to approve the study due to concerns about causing harm to vulnerable children; a lack of familiarity with research involving children; a lack of understanding about the value of children being research participants; and concerns that families receiving palliative care are too vulnerable to participate in research.

Both teams overcame these challenges by anticipating the IRB/REC concerns and providing the committees with detailed applications which included: evidence-based information outlining the benefits of research participation for children^16,24,25^; a detailed distress protocol; an information sheet on support services for parents; and evidence that the application had been developed with user involvement from children. One team found it helpful to offer resources to their ethics committee to learn about conducting research with this population. The ethics committees were more comfortable reviewing the applications when palliative care researchers became members of the committee (US only).

### Navigating gatekeeping in recruitment

Access to child research participants is often mediated or obstructed by adult ‘gatekeepers’. Both teams faced professionals taking a paternalistic stance and aiming to protect vulnerable families at a highly sensitive time. Professionals specialising in working with adults may be unfamiliar with the rights of children to have a voice in matters affecting them and can have a negative opinion of research involving children. Heavy caseloads, inadequate time, competing priorities and research fatigue can also be obstacles. Parents also acted as a barrier to recruitment, denying access to their children in order to shield and protect them from excessive exposure to the parent’s serious illness.

Both teams navigated these challenges by: partnering with clinical agencies as soon as possible; promoting the study as widely as possible, including online; following up on any and all recruitment opportunities, however unlikely; taking a broad definition of life-limiting illness, so that children whose parents are living with life-limiting conditions such as renal failure and cystic fibrosis can be included; minimising the time and work commitment for clinical teams, for example the screening and consent/assent process should be managed by the research team. Other strategies employed were: nurturing practitioners who are supportive of the research; focusing on practitioners who have formed longer term/trusting relationships with families; engaging clinical teams by providing free training/education around this issue and offering to present the final findings of the study with continuing education credits; developing supportive resources including scripts to help recruiters manage parental barriers (see Box 1) and developing short recruitment emails and videos^26^; regularly attending clinical team meetings to identify barriers to recruitment and report back on progress; and developing children’s, young adults and parents’ project advisory groups to support recruitment, data collection and analysis. It is important that professionals and parents are made aware that children derive benefit from taking part in research and appreciate the opportunity to express their opinion about an important issue in their lives.

**Box 1.** Example of guidance provided to professionals recruiting to palliative care research project involving children.

*Thank you for agreeing to help with recruitment to the project. We are looking to recruit children whose parent has a life-limiting illness.* When research involves children, it is not unusual to be worried about the possibility of causing harm or distress. Parents may be concerned about the impact on their child of being involved in research. Below is an example of a way of introducing the project to a parent: *Could I tell you about a project that research colleagues are currently undertaking? Their research is looking at how parental life-limiting illness affects children. They are interested to learn more about this by talking to children whose parents have a life-limiting illness. [Name of researcher] is leading this project and has asked me to talk to parents about the research. Might this be something that you would like to explore further? Could I give you an information sheet and give your contact details to the research team?* Here are some suggestions about how you might provide additional reassurance about the project and answer some frequently asked questions: • Evidence suggests that taking part in research rarely causes harm or additional distress to children. • There is evidence that children appreciate being included in research and having their opinions heard. • Children can obtain comfort from helping other children in a similar situation. • It is rare for children to be included in research in the field of palliative care – but children have a right to be heard, particularly in matters that affect them. • The interviewer is experienced at working with children facing serious parental illness and bereavement and will be sensitive to any potential distress. • The interviewer is happy to meet with parents beforehand and discuss any concerns. • The child will not be given any information beyond what they already know. The purpose of the study is simply to get their perspective on having a parent with a life-limiting illness. • Even if the parents are keen for their child to take part, the child will have the opportunity to refuse and this will be respected. • We have a detailed plan if the child does become distressed in any way or if the child tells us something that is worrying. • We ask the children how the interview was for them (listening closely for distress) and ask who they could talk to after the interview if they felt distressed. We follow up with the families and offer appropriate support after the interview.

## Conclusions

Both teams are committed to the voices of children being heard in palliative care research and believe that their perspectives should inform clinical practice. Denying children the opportunity to participate in research denies them agency and contravenes their human rights. Ethics committees and professional/parental gatekeeping can be barriers to recruitment, and we wanted to share the strategies employed by both teams to overcome these obstacles (Box 2 contains a brief summary of the strategies). These strategies complement other guides to conducting research with children.^1,6–8^ As the challenges of negotiating ethics committees and navigating gatekeeping in recruitment appear to be universal, the strategies should also apply beyond the USA and UK. Our intention is to highlight the facilitators to recruiting children and encourage more palliative care research that includes their unique perspectives.

**Box 2.** Summary of strategies to enhance the participation of children in palliative care research.

*Negotiating ethics committees* • anticipating the IRB/REC concerns and providing the committees with • detailed applications • providing evidence-based information outlining the benefits of research participation for children • providing a detailed distress protocol (an example is available as a Supplemental Material) • providing an information sheet on support services for parents • demonstrating evidence of user involvement from children • providing resources to their ethics committee to learn about conducting research with this population *Navigating gatekeeping in recruitment* • partnering with clinical agencies as soon as possible • promoting the study as widely as possible, including online • following up on all recruitment opportunities, however unlikely • taking a broad definition of life-limiting illness • minimising the time and work commitment for clinical teams, for example the research team taking on all aspects of consent and follow-up • nurturing relationships with practitioners who are supportive of the research • focusing on practitioners who have formed longer term/trusting relationships with families • engaging clinical teams by providing free training/education around this issue and offering to present the final findings of the study with continuing education credits • developing supportive resources including scripts to help recruiters manage parental barriers • developing short recruitment emails and videos for clinical teams • regularly attending clinical team meetings to identify barriers to recruitment • reporting back to the clinical teams on progress • developing children’s, adolescents’ and parents’ project advisory groups to support recruitment, data collection and analysis

## Supplemental Material

sj-pdf-1-pmj-10.1177_02692163221122302 – Supplemental material for Strategies for recruiting the dependent children of patients with a life-limiting illness as research participants Click here for additional data file. Supplemental material, sj-pdf-1-pmj-10.1177_02692163221122302 for Strategies for recruiting the dependent children of patients with a life-limiting illness as research participants by Steve Marshall, Pam Stephenson and Denice Sheehan in Palliative Medicine
