# Supplementary material for: Strategies for recruiting the dependent children of patients with a life-limiting illness as research participants
Source: Palliat Med. 2022 Sep 14;36(10):1570–4. doi: 10.1177/02692163221122302 (PMC9749010; doi:10.1177/02692163221122302)
Supplement: sj-pdf-1-pmj-10.1177_02692163221122302 – Supplemental material for Strategies for recruiting the dependent children of patients with a life-limiting illness as research participants [file sj-pdf-1-pmj-10.1177_02692163221122302.pdf]

Distress Protocol – The SCYP Project

**Pre Interview Explanation**	Explain to the participant that due to the sensitive nature of the research it is possible that they might find some questions / discussions upsetting and difficult to answer. Reiterate to the participant that:<br>• They do not have to answer any questions that they do not want to answer.<br>• They can say that they want to have a break at any time.<br>• If they want to stop the interview, they can do so without giving any reason.<br><br>When speaking to the participant's parent / carer, acknowledge that they too may find involvement upsetting. Explain that there is information available to signpost them to appropriate support.
**During Interview Recognising Distress**	A participant indicates that they are distressed or exhibits behaviours which would indicate distress – crying, becoming quiet, fidgeting, shouting, sudden change in their presenting behaviour, shaking, moving away from the researcher, seeking their parent / carer.
**During Interview Step 1 – Acknowledge Distress**	Stop the interview questions or discussion and acknowledge the distress e.g. 'I can see that talking about this is making you upset…'
**During Interview Step 2 – Offer to Stop the Interview**	Remind the participant that: they may stop for a break, stop the interview if they are finding it too upsetting, or withdraw completely from the study.
**During Interview Step 3 - Review**	• If the participant wishes to continue, carry on with the interview.<br>• If the participant wishes to have a break, offer to give them some time or to return on another day (ensure there are drinks, crayons, paper and other small, age appropriate, activities available for the participant to engage in to help de-escalate their distress).<br>• If the participant does not wish to continue, terminate the interview.
**Post Interview Step 4 – Offer Support**	At the end of the interview, or on terminating the interview, acknowledge again that the participant found the interview distressing.<br>• Inform the participant that their parent / carer will be informed that they have been distressed during the interview.<br>• Talk to the participant's parent / carer about the child / young person's distress.<br>• Talk to the participant and their parent / carer, offer any required support and ensure that they are all comfortable before leaving.<br>• Inform the participant and their parent / carer that they will be contacted within 24 hours by the researcher to ensure that the participant is not unduly distressed and to

	ascertain whether any further support is required.<br>• Provide the parent/carer with the information sheet about support services for anyone facing a bereavement.
**Post Interview**<br>**Step 5 – Feedback – Discuss with senior research team**	After each interview, where the distress protocol has been used, discuss the interview with the Principal Investigator for the research, outlining: the cause of the distress, actions taken, any ongoing concerns, and agree any further action required.
**Post Interview**<br>**Step 6 – follow-up call 24 hours after the interview**	Telephone call 24 hours after the interview to the parent / carer to enquire how the participant is and whether their parent / carer feels they would benefit from external support. Parents/carers will be reminded of the support listed in the information sheet about support services for children/young people facing a bereavement. If the researcher remains concerned about the participant, with the agreement of the parent/carer, they will notify the participant's clinical team, GP or school.<br><br>If there are concerns about the parent / carer's wellbeing during the phone call, they will also be directed to support from their GP or clinical team. They will also be reminded about the information sheet about support services.
**Research Governance**<br>**Step 7 – Record in Research Log / Diary**	Record all instances where the distress protocol has been used in a research log or diary. These anonymised records will be used to inform the development of future studies and topic guides.
